# Molecular Regulation of Heme Oxygenase-1 Expression by E2F Transcription Factor 2 in Lung Fibroblast Cells: Relevance to Idiopathic Pulmonary Fibrosis

**DOI:** 10.3390/biom12101531

**Published:** 2022-10-21

**Authors:** Qinmao Ye, Sarah J. Taleb, Heather Wang, Narasimham L. Parinandi, Daniel J. Kass, Mauricio Rojas, Cankun Wang, Qin Ma, Jing Zhao, Yutong Zhao

**Affiliations:** 1 Department of Physiology and Cell Biology, Dorothy M. Davis Heart & Lung Research Institute, The Ohio State University, Columbus, OH 43210, USA; 2 Department of Medicine, The University of Pittsburgh, Pittsburgh, PA 15213, USA; 3 Department of Internal Medicine, The Ohio State University, Columbus, OH 43210, USA; 4 Department of Bioinformatics, The Ohio State University, Columbus, OH 43210, USA

**Keywords:** heme oxygenase-1/HO-1, gene suppression, transcriptional factor, E2F2, lung fibrosis

## Abstract

Idiopathic pulmonary fibrosis (IPF) is a fatal chronic lung disease. Heme oxygenase-1 (HMOX1/HO-1) is an enzyme that catalyzes the degradation of heme. The role of HO-1 in the pathogenesis of IPF has been studied; however, the molecular regulation of HO-1 and its role in IPF are still unclear. In this study, we found that HO-1 protein levels significantly increased in lung myofibroblasts in IPF patients and in lungs in a murine model of bleomycin-induced lung fibrosis. In addition, we observed that administration of a E2F transcription factor inhibitor elevated HO-1 mRNA and protein levels in lung fibroblasts. Downregulation of E2F2 by siRNA transfection increased HO-1 mRNA and protein levels, while overexpression of E2F2 reduced HO-1 levels. However, overexpression of E2F2 did not alter hemin-induced HO-1 protein levels. Furthermore, modulation of HO-1 levels regulated TGF-β1-induced myofibroblast differentiation without altering the phosphorylation of Smad2/3 in lung fibroblast cells. Moreover, the phosphorylation of protein kinase B (Akt) was significantly upregulated in HO-1-depleted lung fibroblast cells. In summary, this study demonstrated that E2F2 regulates the baseline expression of HO-1, but has no effect on modulating HO-1 expression by hemin. Finally, elevated HO-1 expression contributes to the TGF-β1-induced lung myofibroblast differentiation through the activation of the serine/threonine kinase AKT pathway. Overall, our findings suggest that targeting E2F2/HO-1 might be a new therapeutic strategy to treat fibrotic diseases such as IPF.

## 1. Introduction

Idiopathic pulmonary fibrosis (IPF) is a chronic lung disease with a high mortality that is characterized by aberrant myofibroblast differentiation and extracellular matrix (ECM) accumulation in the lungs. The estimated median survival time for IPF patients is 3 to 5 years, with a high incidence in the elderly population [1]. However, the specific mechanisms of IPF are poorly understood, and lung transplantation is still the most effective approach for IPF treatment [2].

Differentiation of lung fibroblasts into myofibroblasts is a crucial step in the initiation and development of IPF. Transforming growth factor-β1 (TGF-β1) is a significant factor in the induction of myofibroblast differentiation in lung fibrosis, generally thought to be produced by macrophages [3]. TGF-β1 activates TGF-β receptors I and II, and in the canonical TGFβ-Smad2/3 pathway, Smad2 and Smad3 are phosphorylated subsequently and interact with Smad4 to form a complex that translocates into the nucleus to facilitate ECM expression. The expression of α-smooth muscle actin (α-SMA) and increased ECM (collage and fibronectin (FN)) production [4] are markers for myofibroblast differentiation. Understanding the factors that induce myofibroblast differentiation in lung fibroblast cells will be significantly beneficial in revealing the mechanisms of IPF.

Heme oxygenase-1 (HO-1) is an enzyme that catalyzes the reaction of heme catabolism [5]. HO-1 converts heme to carbon monoxide (CO), biliverdin, and ferrous iron, which exerts anti-inflammatory and antioxidant effects [5,6,7,8]. The inducible isoform HO-1 has been reported to be regulated by Nrf2 in response to oxidative stress [9]. Although the pathogenesis of IPF is unclear, some studies suggest that oxidative stress may be involved [9,10,11,12]. It has been reported to play a significant role against inflammatory and oxidant-induced lung injury [4,13]. However, Wang et al. reported that inhibiting HO-1 attenuates rat liver fibrosis [14]. HO-1 levels have been shown to be increased in some pulmonary diseases such as IPF [4,13]; however, Ye et al. have shown that HO-1 levels were lower in macrophages from IPF patients [15]. These discrepancies may be attributed to differences in the molecular mechanisms of HO-1 expression in different cell types. The aim of this study was to identify the regulatory mechanisms of HO-1 expression in lung fibroblasts.

E2F is a family of transcription factors that regulates the cell cycle and DNA synthesis [16]. Several studies have indicated that it is associated with pulmonary fibrosis [17,18,19]. E2F2 is a member of the E2F family and serves as a transcription activator to regulate the cell cycle [20]. An E2F inhibitor, HLM006474, has been found to block E2F1, E2F2, and E2F4 DNA-binding activity [21]. In this study, we showed that HLM006474 significantly increases HO-1 mRNA and protein levels in lung fibroblast cells. In addition, downregulation of E2F2 upregulates HO-1 mRNA and protein levels, while overexpression of E2F2 reduces HO-1 protein levels. Moreover, E2F2 appears to modulate the baseline expression in HO-1, but not hemin-induced HO-1. Finally, HO-1 promotes TGF-β1-induced myofibroblast differentiation, potentially via the AKT signaling pathway but not through the TGF-β-Smad pathway. This study provides evidence showing that E2F2/HO-1 are potential therapeutic targets for treating IPF.

## 2. Materials and Methods

### 2.1. Cell Culture and Reagents

Primary human lung fibroblast cells derived from adult control human subjects (HLF) and IPF patient myofibroblast cells (IPF) were obtained from the Center for Organ Recovery and Education and Lung Transplantation at the University of Pittsburgh. The study was approved by the Institutional Review Board at the University of Pittsburgh (STUDY18100070). The cells were cultured and used at passages 3–6. Human fetal lung fibroblast (Mrc5) cells and normal human fetal lung fibroblast cells (IMR90) were purchased from the American Type Culture Collection (ATCC, Manassas, VA, USA). All human lung fibroblast cells were cultured with Eagle’s Minimum Essential Medium (EMEM) (ATCC) containing 10% fetal bovine serum (FBS), 2 mM L-glutamine, 0.1 mM MEM Eagle Non-Essential Amino Acid Solution (NEAA), and 100 U/mL penicillin-streptomycin. Human small airway epithelial cells (HSAEpC, from Lonza, Walkersville, MD) were cultured in small airway epithelial basal medium (SABM) (Lonza) with SAGM supplements and growth factors (Lonza). Cells were cultured at 37 °C in a 5% CO_2_ incubator. All the cellular treatment experiments were performed in basal media without supplements. HLM006474 (#324461) and hemin were purchased from Sigma (Burlington, MA). HLM006474 and hemin stock solutions were prepared in DMSO and reconstituted in blank media (0.1% v/v) at required doses. Recombinant human TGF-β1 was obtained from R&D systems (Minneapolis, MN, USA). Antibodies for HO-1 and fibronectin (FN) were from ProteinTech (Rosemont, IL, USA). E2F4, E3F6, Smad2/3, and AKT antibodies were from Santa Cruz Biotechnology (Santa Cruz, CA, USA). E2F1, collagen I, and α-SMA antibodies were from Abcam (Waltham, MA, USA). p-Smad2, p-Smad3, p-AKT antibodies were from Cell Signaling (Danvers, MA, USA). β-actin antibody was from Sigma (St. Louis, MO, USA). E2F2 antibody was from Life Technologies (Grand Island, NY, USA). All materials were the highest grade commercially available.

### 2.2. Bleomycin-induced murine model of lung fibrosis

C57BL/6J mice were housed in a specific pathogen-free animal care facility at the Ohio State University. The animal protocol was approved by the Institutional Animal Care and Use Committee (IACUC) at the Ohio State University. Male and female mice aged 8–10 weeks were intratracheally administered bleomycin at a single dose of 2.25 U/kg. After 3 weeks, lung tissues were collected for Western blots and H&E staining.

### 2.3. Western Blots

Protein from cells and mouse lung tissues were extracted using lysis buffer with protease and phosphatase inhibitor (Thermo Fisher Scientific, Waltham, MA, USA). Lysates were sonicated for 12 s on ice and immediately centrifuged at 10,000 rpm at 4 °C for 5 min. Protein assay was performed using the DC protein Assay kit from Bio-Rad Laboratories (Hercules, CA, USA). Then, 15–20 μg of protein was loaded into SDS-PAGE gels (Invitrogen, Carisbad, CA, USA) and transferred to nitrocellulose blotting membrane (0.2 µm, Amersham Protran, Germany). Blots were blocked with 5% nonfat biological grade milk or bovine serum albumin (BSA) in Tris-buffered saline with Tween (TBST, 25 mM Tris-HCl, 137 mM NaCl, and 0.1% Tween 20) for 1 h, then incubated with primary followed by secondary antibodies. Protein expression was detected using an Enhanced Chemiluminescence Detection Kit (Thermo Fisher Scientific).

### 2.4. Plasmid and siRNA/esiRNA Transfection

When cells reached 80–90% confluence, plasmids and siRNA/esiRNA were transfected into the cells. Genjet Plus reagent (SignaGen Laboratories, Rockville, MD, USA) was used for plasmid transfections. PepMute reagent (SignaGen Laboratories) was mixed with an siRNA/esiRNA suspended in PepMute reagent buffer to knock down target genes. All siRNA/esiRNA were purchased from Sigma. The E2F2 plasmid was a gift from Kristain Helin (Addgene plasmid #24226), and HO-1-Myc was obtained from Origene (Rockville, MD, USA).

### 2.5. RNA Extraction, RNA Sequencing, and Quantitative Real-Time PCR

Total RNA was extracted from cells after treatment with an RNA Extraction Kit (Invitrogen). Supermix for RT-PCR kit was used to prepare cDNA (Bio-Rad), and real-time PCR was performed with a SsoFast EvaGreen Supermix kit (Bio-Rad). Primers used for q-PCR were designed based on human mRNA sequences. HO-1 primers: forward 5′- CTCCCAGGGCCATGAACTTT-3′ and reverse 5′- GGTAAGGAAGCCAGCCAAGA-3′. E2F2 primers: forward 5′-AGACTAAGGACTAGAGAGCGA-3′ and reverse 5′-GTCTCGACTGCACCGACTTC-3′. E2F4 primers: forward 5′-TTGAGCCCATCAAGGCAGAC-3′ and reverse 5′-CATGCACTCTCGTGTGGGAT-3′. E2F6 primers forward 5′-TACCCAGTCTCCTCCTGGAC-3′ and reverse 5′-TTTTTGATGGCAGCAGGCCC-3′. All procedures were carried out according to the manufacturer’s instructions. RNA sequencing was performed by The Genomics Shared Resource at the Ohio State University.

### 2.6. Quantification and Statistical Analysis

Immunoblots were quantified by Image J and normalized to β-actin. Comparisons of blot intensities were subjected to statistical analysis using the two-tailed Student’s t-test and ANOVA. Data are expressed as the mean ± SD of triplicate samples from at least three independent experiments. *p* < 0.05 was considered statistically significant.

## 3. Results

### 3.1. HO-1 Protein Levels Are Significantly Increased in Fibrotic Lungs

**Figure 1:**
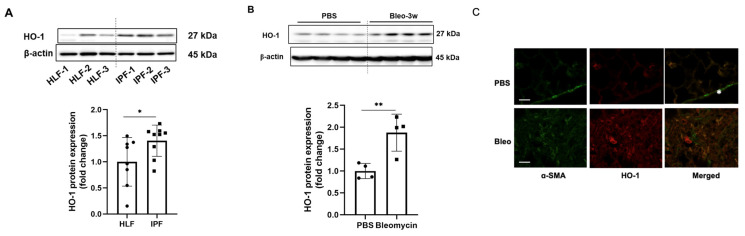
HO-1 protein levels are significantly increased in lung fibrosis. (**A**) Lung fibroblast cells were isolated from the IPF patients and control subjects (HLF). Images shown are representative samples (HLF, *n* = 7; IPF, *n* = 8). Cell lysates were analyzed by immunoblotting with HO-1 and β-actin antibodies. Intensities of HO-1 blots were analyzed by Image J. ** *p* < 0.01, *n* = 7–8. (**B**) C57BL/6 mice were intratracheally given a single dose of bleomycin (2.25 U/kg). After 3 weeks, lung tissue lysates were subjected to Western blotting with HO-1 and β-actin antibodies. Intensities of HO-1 blots were analyzed by Image J. ** *p* < 0.01, *n* = 4. (**C**) Lung tissues were subjected to immunofluorescence staining with antibodies against α-SMA (green) and HO-1 (red). * shows vascular smooth muscle cells as a positive control for α-SMA. Scale bar, 50 μm.

Previous studies have reported that HO-1 protein levels in the lungs were higher in IPF patients [4,13], while they were decreased in lung macrophages isolated from IPF patients [15]. To characterize the expression of HO-1 in lung fibroblasts in IPF, human lung fibroblast cells were isolated from human IPF and control donors. As shown in Figure 1A, HO-1 protein levels were significantly higher in IPF fibroblast cells (IPF) compared with the control subjects (HLF, *p* < 0.01). We then examined HO-1 protein levels in experimental lung fibrosis. Intratracheal injection of a single dose of bleomycin is a well-accepted murine model of IPF [22]. Wild-type mice (C57BL/6) were sacrificed 3 weeks after intratracheal bleomycin or PBS administration, and protein levels of HO-1 in lung tissues were examined. Increased expression of HO-1 was detected in lungs from bleomycin-challenged mice compared with control mice (Figure 1B). Bleomycin-induced lung fibrosis was confirmed by H&E staining (Figure S1). Lung tissues were co-immunofluorescence stained with HO-1 and α-SMA antibodies. Myofibroblasts exhibited higher levels of α-SMA and HO-1 (Figure 1C), indicating that HO-1 increased in myofibroblasts.

### 3.2. E2F Inhibitor Upregulates HO-1 mRNA and Protein Levels

**Figure 2:**
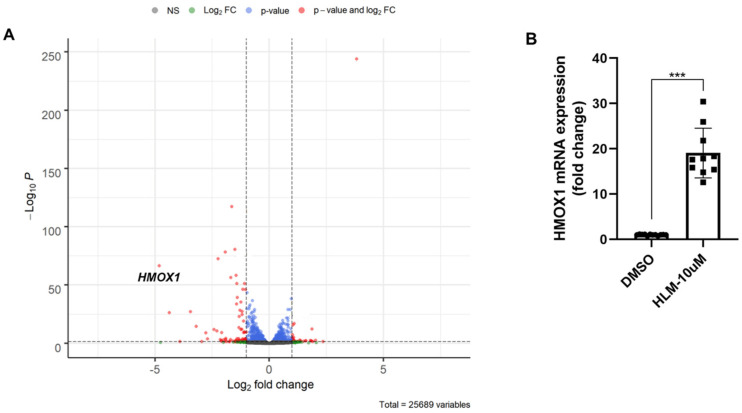
E2F inhibitor upregulates HO-1 mRNA level. (**A**) Primary human lung fibroblast cells were treated with 10 μM E2F inhibitor HLM for 24 h. RNA samples were then collected from cells for RNA sequencing. The result is shown in a volcano plot (*n* = 4). The volcano plot shows differential gene expression in DMSO vs. HLM-treated cells obtained from DESeq2. Significantly increased or decreased genes (red) had a log2-fold change cutoff of 1 and an adjusted *p*-value of <0.05 (Wald test adjusted by Benjamin and Hochberg method). (**B**) Real-time PCR analysis of HO-1 mRNA levels in 10 μM HLM-treated HLF cells (*n* = 10). *** *p* < 0.001, *n* = 10.

**Figure 3:**
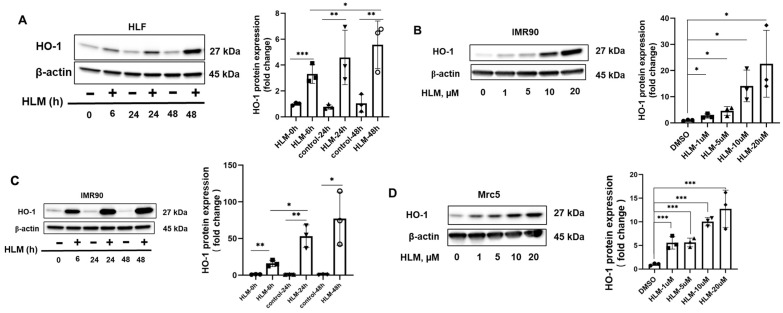
HLM006474 increases HO-1 protein levels. **A)** HLF cells were treated with 10 μM HLM for 0, 6, 24, or 48 h. HO-1 and β-actin levels were analyzed by Western blots (*n* = 3). IMR90 (**B**) and Mrc5 cells (**D**) were treated with 1–20 μM of HLM for 24 h and cell lysates were analyzed by immunoblots with HO-1 and β-actin antibodies. Intensities of HO-1 blots were analyzed by Image J. *** *p* < 0.001, * *p* < 0.05, *n* = 3. (**C**) IMR90 cells were treated with 10 μM of HLM for 0, 6, 24, and 48 h. HO-1 and β-actin levels were examined by Western blots. HO-1 blots were analyzed by Image J. *** *p* < 0.001, ** *p* < 0.01, *n* = 3.

Previous studies have reported that E2F transcription factors are involved in lung fibrosis [17,18,19]. An E2F inhibitor, HLM006474 (HLM), was utilized to investigate the role of E2F in primary HLF. HLM006474 was found to inhibit E2Fs binding to DNA, thereby reducing E2F transcriptional activity. HLF cells were treated with HLM (10 μM) for 24 h, followed by extraction of RNA for sequencing (RNA-seq). HO-1 mRNA levels were significantly increased in HLM-treated HLF cells (Figure 2A). Subsequently, real-time PCR was used to confirm that HLM increased HO-1 expression in HLF cells (Figure 2B). We then examined HO-1 protein levels by immunoblotting. When HLF cells were treated with 10 μM HLM at different time points, we found that the HLM significantly upregulated HO-1 protein levels in a time-dependent manner (Figure 3A). HO-1 protein levels were also significantly increased by HLM in a time- and dose-dependent manner in IMR90 cells (Figure 3B,C). The effect of HLM on the upregulation of HO-1 protein levels was confirmed in another lung fibroblast cell line Mrc5 in a dose-dependent manner (Figure 3D). In addition to lung fibroblasts, HLM treatment significantly elevated HO-1 protein expression in human lung small airway epithelial cells (HASEpC) (Figure S2). Taken together, these data suggested that E2F transcription factors suppressed HO-1 expression.

### 3.3. E2F2 Suppresses the Baseline Expression of HO-1

**Figure 4:**
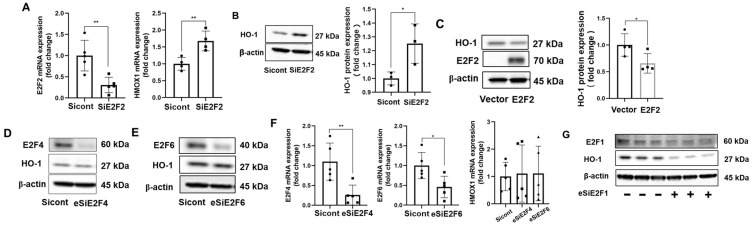
E2F2 suppresses HO-1 expression. (**A**) IMR90 cells were transfected with E2F2 siRNA for 72 h. *E2F2* and *HO-1* mRNA levels were examined by real-time PCR. ** *p* < 0.01, *n* = 4. (**B**) Protein levels of HO-1 and β-actin were analyzed by immunoblots. Intensities of HO-1 blots were analyzed by Image J. * *p* < 0.05, *n* = 3. (**C**) E2F2 plasmid was transfected into IMR90 cells for 48 h, and cell lysates were analyzed by western blots with HO-1 and β-actin antibodies. Intensities of HO-1 blots were analyzed by Image J. * *p* < 0.05, *n* = 4. (**D**,**E**) E2F4 and E2F6 were knocked down in IMR90 cells by esiRNA for 72 h, and E2F4, E2F6, HO-1, and β-actin levels were analyzed by Western blots (*n* = 2). (**F**) After knockdown of E2F4 and E2F6 in IMR90 cells for 72 h, mRNA expression of *E2F4*, *E2F6*, and *HO-1* were examined by real-time PCR (*n* = 5). (**G**) esiRNA was used to downregulate expression of E2F1; protein expression of HO-1, E2F1 and β-actin were analyzed by immunoblots (*n* = 3).

Given that the expression of HO-1 was upregulated by an E2F inhibitor, we further investigated which member of the E2F family was involved. E2F1, E2F2, E2F4, and E2F6 expression was knocked down in IMR90 cells by siRNA/esiRNA transfection. Real-time PCR demonstrated that E2F2 knockdown increased HO-1 mRNA expression (Figure 4A). HO-1 protein levels were also significantly increased in the absence of E2F2 in IMR90 cells (Figure 4B). Next, an E2F2 plasmid was transfected into cells to overexpress E2F2 in IMR90 cells. Reduced HO-1 protein levels were shown by Western blots (Figure 4C). Knockdown of E2F4 and E2F6 had no effects on HO-1 mRNA and protein expression (Figure 4D–F). However, when the expression of E2F1 was silenced, HO-1 protein levels significantly decreased (Figure 4G). Therefore, these findings suggested that E2F1, E2F4 and E2F6 did not downregulate HO-1 expression and that only E2F2 suppressed the baseline HO-1 transcription in human lung fibroblast cells.

**Figure 5:**
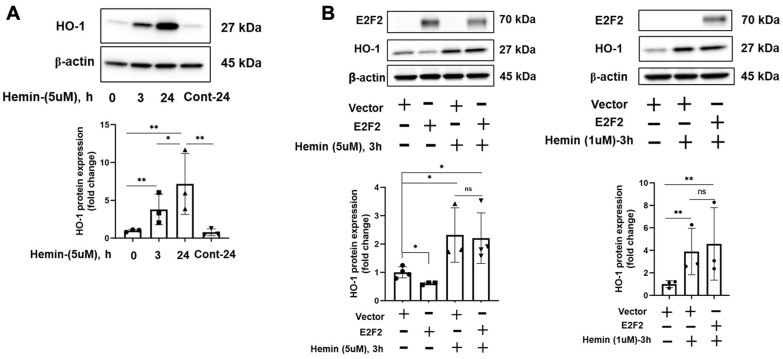
E2F2 does not affect hemin-induced HO-1 expression. (**A**) IMR90 cells were treated with 5 μM hemin for 0, 3, or 24 h. HO-1 protein expression was analyzed by Western blots. Intensities of HO-1 were analyzed by Image J. ** *p* < 0.01, * *p* < 0.05, *n* = 3. (**B**) IMR90 cells were transfected with E2F2 plasmid for 48 h, then treated with 1 or 5 μM hemin for 3 h. Cell lysates were analyzed by Western blots with HO-1, E2F2, and β-actin antibodies. Intensities of HO-1 bands were analyzed by Image J. ** *p* < 0.01, * *p* < 0.05, *n* = 3–4.

To determine whether E2F2 suppresses HO-1 expression under oxidative stress, hemin, a HO-1 inducer [23], was utilized to induce the expression of HO-1 in human lung fibroblast cells (Figure 5A). We transfected IMR90 cells with *E2F2* plasmid for 48 h, followed by treatment with 1 and 5 μM of hemin for an additional 3 h. Unexpectedly, E2F2 did not modulate HO-1 expression induced by hemin at any given dose (Figure 5B).

### 3.4. HO-1 Promotes TGF-β1-Induced Myofibroblast Differentiation without Altering Phosphorylation of Smad2/3

**Figure 6:**
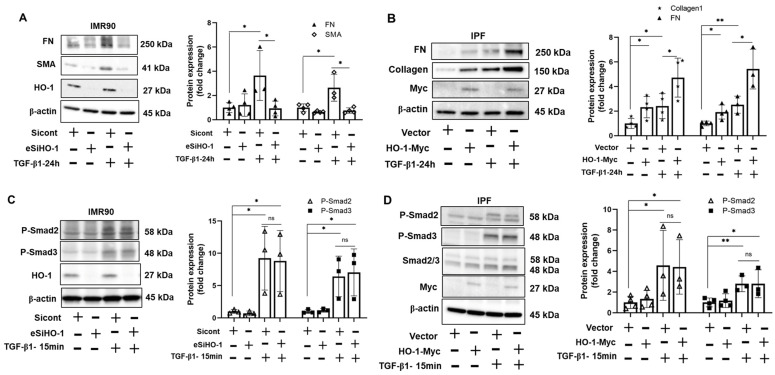
HO-1 promotes TGF-β1-induced myofibroblast differentiation. IMR90 cells were transfected with HO-1 esiRNA for 72 h, then treated with TGF-β1 (10 ng/mL) for 24 h (**A**) and 15 min (**C**). Cell lysates from 24 h of TGF-β1 stimulation were analyzed by Western blots with fibronectin (FN), α-smooth muscle actin (SMA), HO-1, and β-actin antibodies and lysates from 15 min of stimulation were analyzed by Western blots with p-Smad2/3, HO-1 and β-actin antibodies. Intensities of blots were analyzed by Image J. * *p* < 0.05, *n* = 3. IPF cells were overexpressed with HO-1-Myc plasmid for 48 h, then treated with TGF-β1 for 24 h (**B**) and 15 min (**D**). FN, collagen, Myc tag, and β-actin antibodies were used to analyze protein levels by immunoblots lysate from TGF-β1 24-h treatment, and Smad2/3, p-Smad2/3, Myc and β-actin antibodies were used to examine protein levels from TGF-β1 15-min treatment. Intensities of p-Smad2 and p-Smad3 bands were analyzed by Image J. ** *p* < 0.01, * *p* < 0.05, *n* = 3.

To investigate the role of HO-1 in TGF-β1-induced myofibroblast differentiation, we first studied whether TGF-β1 modulates HO-1 expression. In response to TGF-β1 treatment, HO-1 protein levels did not significantly change in human lung fibroblast cells (Figure S3A,B). We then knocked down or overexpressed HO-1 in human lung fibroblast cells with subsequent TGF-β1 stimulation. TGF-β1-induced FN and α-SMA were reduced in the absence of HO-1 (Figure 6A). Meanwhile, overexpression of HO-1 promoted FN and collagen 1 production (Figure 6B). However, TGF-β1-induced phosphorylation of Smad2/3 was not affected by overexpression nor knockdown of HO-1 (Figure 6C,D). These data indicated that HO-1 enhanced TGF-β1-induced myofibroblast differentiation in human lung fibroblast cells; however, it may be due to a process other than the canonical TGF-β signaling pathway.

### 3.5. HO-1 Regulates Phosphorylation of AKT

**Figure 7:**
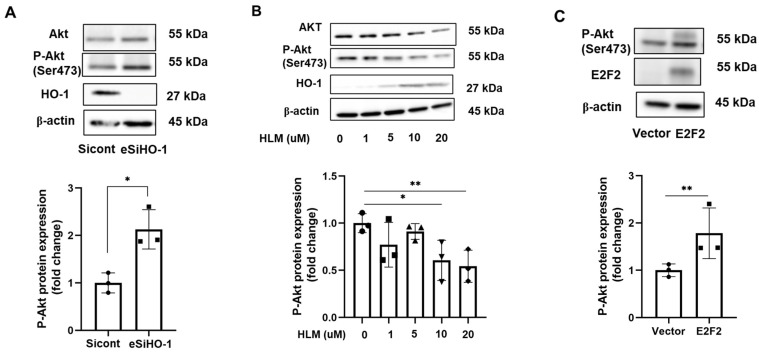
HO-1 regulates phosphorylation of AKT. (**A**) esiRNA was used to knockdown HO-1 levels in IMR90 cells for 72 h, then HO-1, P-AKT, AKT, and β-actin protein levels were quantitated by immunoblots. Intensities of P-AKT bands were normalized to β-actin and quantified by Image J. * *p* < 0.05, *n* = 3. (**B**) IMR90 cells were treated with 1–20 μM HLM for 24 h. Protein expression of AKT, P-AKT, HO-1, and β-actin were quantitated by Western blots. Intensities of P-AKT bands were normalized to β-actin and quantified by Image J. * *p* < 0.05; ** *p* < 0.01, *n* = 3. (**C**) E2F2 plasmid was transfected into IMR90 cells for 48 h, and cell lysates were quantitated by Western blots with P-AKT, E2F2, and β-actin antibodies. Intensities of P-AKT bands were normalized to β-actin and quantified by Image J. ** *p* < 0.01, *n* = 3.

The serine/threonine kinase AKT pathway has been reported to regulate the pathogenesis of pulmonary fibrosis [24,25,26,27]. We found that phosphorylation of AKT (Ser473) increased when HO-1 was knocked down in IMR90 cells (Figure 7A). Furthermore, administration of E2F inhibitor (HLM) increased HO-1 and reduced phosphorylation of AKT in a dose-dependent manner (Figure 7B). Overexpression of E2F2 also upregulated phosphorylation of AKT in human lung fibroblast cells (Figure 7C). Taken together, these data indicated that E2F2 regulation of HO-1 may suppress phosphorylation of AKT, and is likely involved in HO-1-mediated myofibroblast differentiation.

## 4. Discussion

Abnormal expression of HO-1 is involved in human disorders including IPF. In this study, we investigated the molecular mechanisms involved in the regulation of HO-1 expression in lung fibroblasts. HO-1 expression was elevated in lung fibroblasts in IPF and in fibrotic lungs in experimental lung fibrosis. We also found that E2F inhibitor upregulated HO-1 mRNA and protein levels. Thus, we speculate that E2F2 negatively modulates the transcription of HO-1. Interestingly, E2F2 is not involved in regulating HO-1 in response to oxidative stress, indicating that E2F2 participates only in regulating the baseline expression of HO-1. Furthermore, we showed that HO-1 expression is essential for TGF-β1-induced ECM accumulation in lung fibroblasts without altering the TGF-β canonical pathway. The mechanisms by which HO-1 regulates TGF-β1-induced myofibroblast differentiation have not been revealed. Our findings suggest that phosphorylation of AKT protein is a downstream HO-1 signal.

The impact of HO-1 in lung fibrosis is controversial. A previous report has suggested that an HO-1 inhibitor (Zn-deuteroporphyrin-IX-2,4-bisethylene glycol, ZnBG) ameliorates bleomycin-induced pulmonary fibrosis [28], while another study suggested that overexpression of HO-1 by adenoviral vector reduces experimental lung fibrosis [29]. In this study, we showed that HO-1 promoted TGF-β1-induced myofibroblast differentiation, suggesting that the baseline expression of HO-1 is necessary for fibroblast differentiation. TGF-β1-mediated lung fibroblast activation and differentiation to myofibroblasts are implicated in the development of lung fibrosis [4]. Thus, our study supports that activation of HO-1 may play a role in the pathogenesis of lung fibrosis. It is difficult to conclude that HO-1 is a solely pro-fibrotic signaling molecule as it also exerts anti-oxidant properties and may protect lungs from injury and fibrogenesis. The progression of lung fibrosis involves repetitive lung injury, repair, fibroblast activation, and lung architecture disruption. It is likely that HO-1 plays different roles at different stages of fibrosis development. In the initial stages, induction of HO-1 by oxidative stress may have cytoprotective effects; however, during the abnormal lung repair and remodeling, HO-1 may promote TGF-β signaling and myofibroblast differentiation. Thus, development of a conditional HO-1 knockout mouse will be useful to investigate the role of HO-1 in lung fibrosis.

Several studies have been identified that the transcription factor NF-E2-related factor 2 (Nrf2) plays a significant role in the regulation of HO-1 expression under oxidative stress [30,31]. In the nucleus, Nrf2 binds to anti-oxidant response elements (ARE), a domain that mediates transcriptional activation of genes including the HO-1 in responding to oxidative stress [31,32,33]. In the current study, we demonstrated that E2F2 suppressed the baseline transcription of HO-1 in lung fibroblast cells. Similar to lung fibroblasts, HLM006474 also increased HO-1 levels in lung epithelial cells, suggesting that E2F2 regulates baseline expression of HO-1 in different lung cell types. In contrast to the effect of E2F2, we found that downregulation of E2F1 reduced HO-1 levels. It has been shown that E2F members play distinct roles in the regulation of gene expression [34]. It is possible that E2F1 and E2F2 compete for the E2F binding motif on the HO-1 promotor. As HLM006474 inhibits both E2F1 and E2F2, HLM006474-induced HO-1 expression is through inhibition of E2F2. This study focused on the molecular regulation of HO-1 expression. Our future studies will investigate E2F2 regulation on HO-1 transcription and if E2F2 binds to the HO-1 promoter directly. We found that TGF-β1 did not alter HO-1 protein levels within 24 h in lung fibroblast cells; however, a previous study reported that TGF-β1 regulates HO-1 mRNA and protein in A549 cells [35]. The difference suggests that baseline HO-1 is likely to be influenced by different mechanisms in response to TGF-β1 by different cell types.

The relationship between the AKT pathway and HO-1 has been reported. Most studies have focused on the role of AKT in the regulation of HO-1 expression [31,36,37,38]. It is generally believed that the AKT pathway regulates HO-1 expression. It has been shown that inhibition of the PI3K/AKT pathway reduced HO-1 expression by modulating Nrf2 expression and activation [37]. We observed that HO-1 regulated AKT phosphorylation. It is unclear by which catalytic by-products, HO-1 regulates AKT. One of by-products of HO-1, CO, has been shown to regulate the GSK3β/PI-3K/AKT pathway [39]. It is possible that HO-1-mediated AKT phosphorylation is initiated through CO production in lung fibroblasts. Activation of AKT has been shown to inactivate FoxO3 transcription factor in myofibroblasts [40]. We will investigate if HO-1 regulates FoxO3 transcriptional activity in future studies.

## Data Availability

Not applicable.

## Supplementary Materials

The following supporting information can be downloaded at: https://www.mdpi.com/article/10.3390/biom12101531/s1, Figure S1: Bleomycin induces lung fibrosis. Figure S2: HLM006474 increases HO-1 levels in HSAEpCs. Figure S3: HMOX1 upregulation is not through TGF-β1 stimulation.
