# Supplementary material for: Molecular Regulation of Heme Oxygenase-1 Expression by E2F Transcription Factor 2 in Lung Fibroblast Cells: Relevance to Idiopathic Pulmonary Fibrosis
Source: Biomolecules. 2022 Oct 21;12(10):1531. doi: 10.3390/biom12101531 (PMC9599643; doi:10.3390/biom12101531)
Supplement: Supplementary file 1 [file biomolecules-12-01531-s001.zip › biomolecules-1868316-supplementary.pdf]

Supplemental materials

Molecular Regulation of Heme Oxygenase 1 Expression by E2F Transcription Factor 2 in Lung Fibroblast Cells: Relevance to Idiopathic Pulmonary Fibrosis

Qinmao Ye [1], Sarah J Taleb [1], Heather Wang [1], Narasimham L Parinandi [1], Daniel J Kass [2], Mauricio Rojas [3], Cankun Wang [4], Qin Ma [4], Zhao Jing [1,3], Zhao Yutong [1,3#]

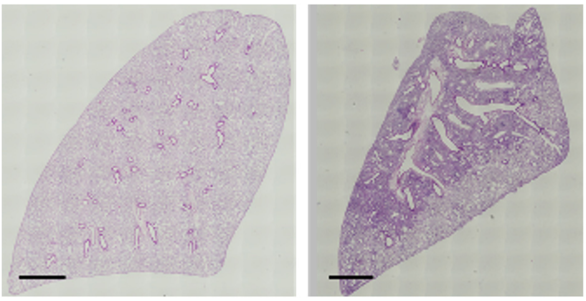

Supplemental Figure S1. Bleomycin indues lung fibrosis. C57BL/6 mice were intratracheal injection with bleomycin. After 3 weeks, lung tissues were subjected to H&E staining. Scale bar, 1 mm.

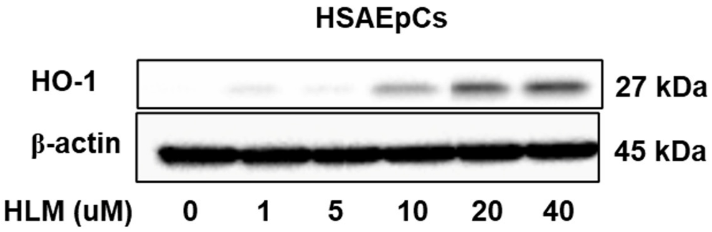

Supplemental Figure S2. HLM006474 increases HO-1 levels in HSAEpCs. HSAEpCs were treated with HLM in different concentrations for 24 hours and HO-1 and β-actin protein levels were analyzed by immunoblotting (n=2).

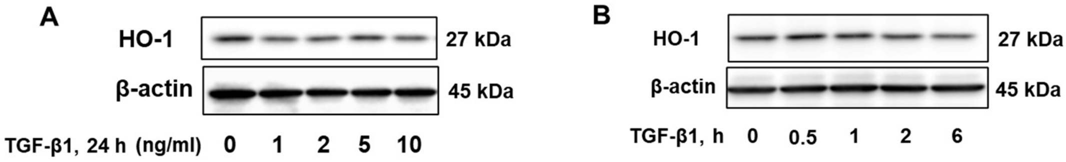

Supplemental Figure S3. HMOX1 upregulation is not through TGF-β1 stimulation. Human lung fibroblast cells (CCL-210) were stimulated with TGF-β1 in different doses **(A)** and time points **(B).** Cell lysates were examined by immunoblotting with HO-1 and β-actin antibodies (n=3).
